# Depletion of B cells rejuvenates the peripheral B‐cell compartment but is insufficient to restore immune competence in aging

**DOI:** 10.1111/acel.12959

**Published:** 2019-05-06

**Authors:** Irit Avivi, Simona Zisman‐Rozen, Shulamit Naor, Isabelle Dai, David Benhamou, Gitit Shahaf, Hilla Tabibian‐Keissar, Noemie Rosenthal, Aviya Rakovsky, Ammuri Hanna, Arik Shechter, Eli Peled, Noam Benyamini, Ekaterina Dmitrukha, Iris Barshack, Ramit Mehr, Doron Melamed

**Affiliations:** ^1^ Department of Hematology Tel Aviv Sourasky Medical Center Tel Aviv Israel; ^2^ Sackler Medical School Tel‐Aviv University Tel Aviv Israel; ^3^ Department of Immunology Faculty of Medicine Technion‐Israel Institute of Technology Haifa Israel; ^4^ The Mina and Everard Goodman Faculty of Life Sciences Bar‐Ilan University Ramat‐Gan Israel; ^5^ Department of Pathology Sheba Medical Center Ramat Gan Israel; ^6^ Department of Family Medicine Technion Faculty of Medicine Clalit Health Services and Neuro‐urology Unit RAMBAM Medical Center Haifa Israel; ^7^ Orthopedic Division Rambam Health Care Campus Haifa Israel; ^8^ Department of Hematology RAMBAM Medical Center Haifa Israel

**Keywords:** aging, B cell depletion, B cell rejuvenation, B cell repertoire, infections prevalence, immunity and severity

## Abstract

Aging is associated with increasing prevalence and severity of infections caused by a decline in bone marrow (BM) lymphopoiesis and reduced B‐cell repertoire diversity. The current study proposes a strategy to enhance immune responsiveness in aged mice and humans, through rejuvenation of the B lineage upon B‐cell depletion. We used hCD20Tg mice to deplete peripheral B cells in old and young mice, analyzing B‐cell subsets, repertoire and cellular functions in vitro, and immune responsiveness in vivo. Additionally, elderly patients, previously treated with rituximab healthy elderly and young individuals, were vaccinated against hepatitis B (HBV) after undergoing a detailed analysis for B‐cell compartments. B‐cell depletion in old mice resulted in rejuvenated B‐cell population that was derived from de novo synthesis in the bone marrow. The rejuvenated B cells exhibited a "young"‐like repertoire and cellular responsiveness to immune stimuli in vitro. Yet, mice treated with B‐cell depletion did not mount enhanced antibody responses to immunization in vivo*,* nor did they survive longer than control mice in "dirty" environment. Consistent with these results, peripheral B cells from elderly depleted patients showed a "young"‐like repertoire, population dynamics, and cellular responsiveness to stimulus. Nevertheless, the response rate to HBV vaccination was similar between elderly depleted and nondepleted subjects, although antibody titers were higher in depleted patients. This study proposes a proof of principle to rejuvenate the peripheral B‐cell compartment in aging, through B‐cell depletion. Further studies are warranted in order to apply this approach for enhancing humoral immune responsiveness among the elderly population.

## 1 INTRODUCTION

Elderly individuals are at increased risk to develop infections, which results in significant morbidity and mortality, accounting for 9% of deaths in elderly subjects (Gibson et al., 2009; Heron & Smith, 2007). Attempts to reduce infection rates by employing vaccinations have only limited success due to the decline in immune system function (Goodwin, Viboud, & Simonsen, 2006; Weinberger, Herndler‐Brandstetter, Schwanninger, Weiskopf, & Grubeck‐Loebenstein, 2008). Efforts to improve vaccine efficacy by refining antigen delivery have also failed to provide the desirable immune protection (Levine & Sztein, 2004; Zheng, Switzer, Marinova, Wansley, & Han, 2007). Hence, novel technologies that target the elderly patient immune system and enhance its responsiveness to vaccinations and pathogens, thereby overcoming the immunodeficiency associated with aging, are required.

Among the most promising interventions in recent years, with demonstrated rejuvenating capacity in mouse models, is the removal of "old" tissues or cells ("Breakthrough of the year, The runners‐up," *Science* 2011). Indeed, when applying this approach in the hematopoietic system, we have demonstrated that removal of "old" B cells reverses B‐cell senescence through reactivation of B lymphopoiesis in the bone marrow (BM) of aged mice (Keren et al., 2011). Similar outcomes have also been reported for other tissues (Chang et al., 2015; Jeon et al., 2017). Considering that senescence of the B lineage is reversible and subjected to homeostatic regulation (Keren et al., 2011; Melamed & Scott, 2012; Shahaf, Zisman‐Rozen, Benhamou, Melamed, & Mehr, 2016), the current study tested whether this new paradigm can be translated to enhance immune response in elderly individuals that have been treated for B‐cell malignancies by transient B‐cell depletion. We show here that B‐cell depletion in both elderly mice and humans rejuvenates the peripheral B‐cell compartments both phenotypically and functionally, through the induction of de novo B lymphopoiesis. However, we found that B‐cell rejuvenation by itself is insufficient to significantly enhance responsiveness to vaccination in aged mice and humans and to prolong survival of old mice.

## 2 METHODS

### 2.1 Mice, B‐cell depletion, and immunizations

Mice being used, 10–12 weeks (young) or at 20–24 months (old), were Balb/c or hCD20Tg Balb/c (expressing the human CD20 molecule on surface of B lineage cells) (Ahuja et al., 2007). To deplete B cells in vivo hCD20Tg mice were injected intraperitoneal with purified monoclonal mouse anti‐hCD20 (clone 2H7) antibodies at 1 mg/mouse as described (Ahuja et al., 2007). Depletion was confirmed by flow cytometric analysis of peripheral blood cells 3 days later. For inducible ablation of recombination activating gene 2 (RAG‐2), we used Rag‐2^fl/fl^ mice, (Hao & Rajewsky, 2001) crossed with Mx‐cre transgenic mice (Berg et al., 1995), enabling ablation of the floxed alleles upon in vivo administration of poly(I)‐poly(C). Immune challenging of old, B‐cell‐depleted mice was conducted 65 days after depletion, when reconstitution of the peripheral compartment was reached (Shahaf et al., 2016). Further details on immunization and treatments of mice are detailed in Appendix S1.

### 2.2 Analysis of BrdU incorporation and mathematical modeling

B‐cell depletion in young and old mice was performed (Shahaf et al., 2016). Mice were analyzed for BrdU incorporation 65 days after depletion. At that point, mice were injected intraperitoneal with BrdU. BM and spleen cells were isolated and stained with anti‐BrdU antibody using the BrdU Flow Kit (BD Biosciences; Shahaf et al., 2016; detailed in the Appendix S1).

### 2.3 B‐cell purification, stimulations, and flow cytometry

Detailed in the Appendix S1.

### 2.4 Antibody quantification and IgH repertoire

Quantification of mouse or human IgM in supernatants (Edry, Azulay‐Debby, & Melamed, 2008), and quantification of IgG specific for OVA, HSA, or BSA in mouse sera (Seagal, Leider, Wildbaum, Karin, & Melamed, 2003) were performed by ELISA.

For mouse splenic B‐cell IgH repertoires, we used high‐throughput sequencing. Human peripheral blood repertoires were investigated by spectratyping using DNA extracted from peripheral blood mononuclear cells (Wu, Kipling, & Dunn‐Walters, 2015; detailed in the Appendix S1).

### 2.5 Statistical analysis

Confidence intervals for diversity and similarity analysis were calculated as described (Tabibian‐Keissar et al., 2016). Statistical significance of differences between groups was calculated by ANOVA. Statistical analysis for the Kaplan–Meier survival plots was done using the log‐rank test.

### 2.6 Patient selection and antibody responses to hepatitis B vaccine (HBV)

A prospective clinical trial was designed to evaluate the humoral response to anti‐HBV vaccine in B non‐Hodgkin lymphoma (B‐NHL) elderly patients, previously treated with rituximab B‐cell depletion therapy (the study group), versus elderly and young healthy volunteers. The study was approved by IRB 3097: NCT00863187.

#### 2.6.1 Inclusion criteria

B‐cell NHL patients, aged ≥55 years, serologically negative for HBV (HBS antigen and anti‐HB Core/HBS/HBe antibodies were negative), who completed R‐CHOP or R‐CVP therapy (rituximab in combination with cyclophosphamide, oncovin, prednisone, with vs. without doxorubicin, respectively) 6–60 months earlier, and whose disease has not progressed since then. Elderly healthy volunteers, aged ≥55 years, serologically negative for HBV, who have never been treated with rituximab, with no autoimmunity. Young healthy volunteers, aged 18–35 years old, serologically negative for HBV, who have never been treated with rituximab, with no autoimmunity. B‐NHL rituximab‐treated patients were required to fulfill the following laboratory parameters suggestive for B‐cell reconstitution, before being vaccinated: B‐cell frequency has already reached ≥75% of the normal range (1%–7% of leukocytes), and/or B‐cell percentages in peripheral blood remained constant for at least two measurements, 3 months apart, blood IgG level was >550 mg/dl, a significant (≥50%) in vitro B‐cell responsiveness to mitogenic stimulations (LPS and CpG), measured by antibody production. B‐NHL patients who fulfilled these criteria, and all healthy participants were vaccinated with three doses of HBV vaccine (Recombivax HB, Engerix‐B, GSK), with boosts administered 1 and 5 months after the first dose (Mast et al., 2006) ("The Selection and Use of Essential Medicines," 2015). A serological test, measuring the humoral response to vaccination, was performed at 4–6 weeks after the last vaccine dosage. Anti‐HBV antibody titers higher than 10 IUμ/L were considered a positive response to HBV vaccination (Jack, Hall, Maine, Mendy, & Whittle, 1999). NHL patients whose B cells did not fulfill the minimal criteria for "B‐cell recovery" (as specified above) were excluded from vaccination.

### 2.7 Biostatistics of the clinical study

The data were analyzed by using SPSS software version 15, with a significance level of *p* < 0.05. A comparison of the laboratory and clinical parameters mentioned above was performed by using a nonparametric ANOVA test (the Kruskal–Wallis test). The relationships between quantifiable laboratory measurements and age were tested by the Mann–Whitney nonparametric test.

## 3 RESULTS

### 3.1 Reconstitution of peripheral B cells after depletion originates from de novo generation in the BM

**Figure 1:**
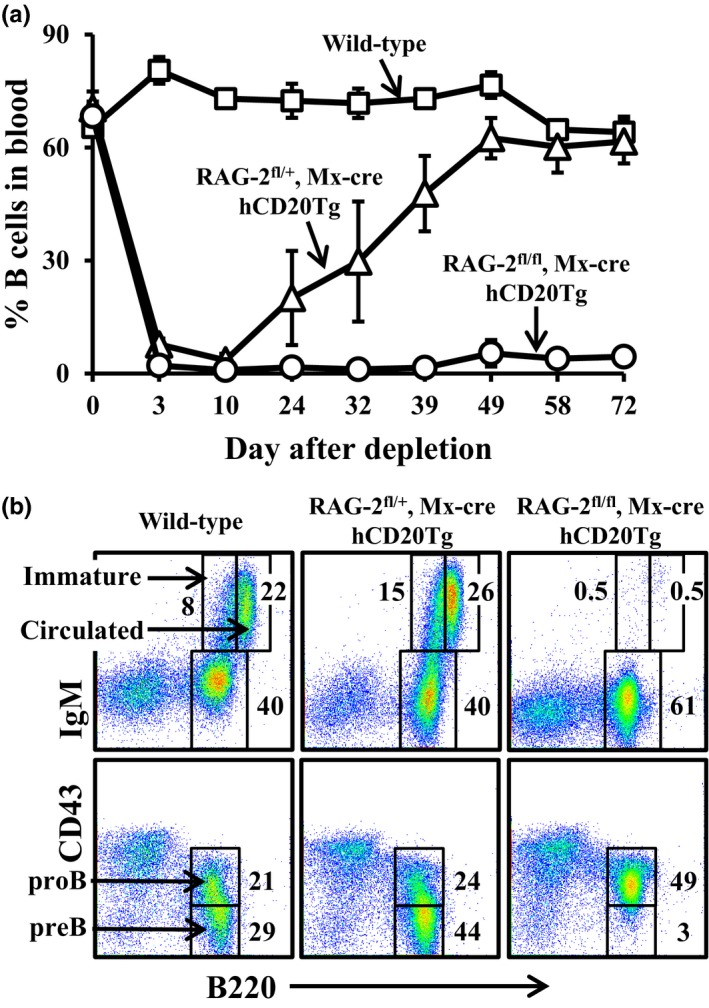
B‐cell reconstitution following depletion occurs through de novo B lymphopoiesis in the BM of old mice. Mice with the indicated genetic backgrounds were injected with poly(I)(C) to ablate RAG‐2‐floxed alleles, followed by injection of anti‐hCD20 antibodies to deplete peripheral B cells, as described in the methodology section. (a) After B‐cell depletion, mice were followed temporally for B‐cell return by measuring frequencies of B cells (B220+/CD19+) in peripheral blood. The results are expressed as mean ± *SE* for four mice in each group. (b) Bone marrow cells of the mice were analyzed for B‐cell populations by flow cytometry 81 days after depletion. Shown are representative results from four mice in each group

B‐cell depletion in human patients and in mice is followed by gradual reconstitution of the peripheral B‐cell compartment. In old mice, we have previously shown that B‐cell depletion reactivates B lymphopoiesis in the BM (Keren et al., 2011). However, reconstitution may also be derived from homeostatic proliferation of residual B cells in the periphery (Yanes, Gustafson, Weyand, & Goronzy, 2017). While generation in the BM gives rise to a new, diverse young‐like B‐cell population, homeostatic proliferation leads to expansion of residual selected clones and oligomonoclonality (Yanes et al., 2017). To study the reconstituted peripheral B‐cell compartment in old mice, we have first determined their regenerative source. To do so, we generated mice that are homozygous for a RAG2‐floxed allele and are transgenic to Mx‐cre and to human CD20 (hCD20) (RAG‐2^fl/fl^/Mx‐cre/hCD20Tg). In these mice, we ablated both RAG‐2 alleles by treatment with poly (I)(C), thus aborting V(D)J recombination and blocking B lymphopoiesis in the BM at the pro‐B stage (Hao & Rajewsky, 2001). Subsequently, we depleted peripheral B cells by injecting anti‐hCD20 antibodies (Ahuja et al., 2007). We then followed the kinetics of B‐cell return in individual mice for 72 days by staining peripheral blood samples, in comparison with wild‐type and to RAG‐2^fl/+^/Mx‐cre/hCD20Tg mice (which carry one nonfloxed RAG‐2 allele and thus allow unaltered B lymphopoiesis after B‐cell depletion). The results in Figure 1 show high efficiency of depletion in peripheral blood of aged hCD20Tg mice, as on day 3 all mice that are transgenic for hCD20 show more than 90% reduction in B‐cell numbers (Figure 1a). However, while frequencies of B cells did not increase with time in the RAG‐2^fl/fl^/Mx‐cre/hCD20Tg (up to 72 days after depletion), the frequency of B cells in peripheral blood of RAG‐2^fl/+^/Mx‐cre/hCD20Tg mice gradually increased, reaching their original number on day 49. Importantly, the reconstituting cells in the RAG‐2^fl/+^/Mx‐cre/hCD20Tg group were newly generated, as reflected by the high proportion of cells expressing the early developmental marker CD93 (AA4.1) in the peripheral blood (Figure S1). Finally, to confirm that B lymphopoiesis was aborted in the RAG‐2^fl/fl^/Mx‐cre/hCD20Tg mice upon ablation of RAG‐2, we analyzed BM cells on day 81 for the following B‐cell populations: pro‐B (B220+/CD43+/IgM−), pre‐B (B220+/CD43−/IgM−), immature (B220+/IgM+), and circulated mature (B220^high^/IgM+). Figure 1b shows a developmental block at the pro‐B stage in the BM of RAG‐2^fl/fl^/Mx− cre/hCD20Tg mice, as essentially no pre‐B (lower panel), immature and circulated mature B cells (upper panel) were detected. In contrast, B lymphopoiesis was not altered in control RAG‐2^fl/+^/Mx− cre/hCD20Tg mice. These results lead us to conclude that B‐cell reconstitution after depletion originates from de novo B lymphopoiesis in the BM and not from homeostatic proliferation of residual B cells.

The main effect of depletion seems to be to reduce the number of mature recirculating B cells in the BM (Figures S3 and S4), thus potentially freeing more niches for new B‐cell development. Indeed, BrdU labeling results (Figure S5) show that in old B‐cell‐depleted mice, more immature B cells become BrdU+ by the second day of labeling than in nondepleted mice. However, one should never draw conclusions regarding, for example, proliferative capacity from the slopes of BrdU labeling curves alone, as these slopes can be made steeper by more rapid turnover as well, that is, increased input of labeled cells or even increased output (death or exit to other compartments). The faster the previous (unlabeled) cells leave, the faster the fraction of labeled cells increases. Thus, mathematical modeling is always necessary in order to interpret BrdU—and any other type of labeling—kinetics experiments, as has been elegantly demonstrated in the past (Asquith, Debacq, Macallan, Willems, & Bangham, 2002; Borghans & Boer, 2007).

Fitting our mathematical model to the BrdU labeling results (Figure S6) reveals that the differentiation rate from the proliferating subpopulation into the immature subset, oe, is higher in old‐depleted mice than in the control mice (Table S1 and Figure S6). This difference is not significant, as the confidence intervals overlap, but the trend is clear. This result is in line with the conclusion that reconstitution of BM B‐cell numbers after depletion in old mice is due to increased generation of new B cells. In addition, the rate of transitional B‐cell differentiation into the splenic mature B‐cell subset was significantly higher in old‐depleted mice than in either old or young control mice (Table S1 and Figure S6), which supports our conclusion. Interestingly, however, the rate of immature cell differentiation into transitional B cells, i_t, was lower in old mice than in young mice, such that overall B‐cell export from the BM to the spleen was lower in old mice. No differences in this rate were found between old and old B‐cell‐depleted mice, indicating that the depletion does not compensate for this age‐related change in B‐cell maturation.

### 3.2 Reconstituted B cells display a young‐like population dynamic, repertoire, and cellular responsiveness

**Figure 2:**
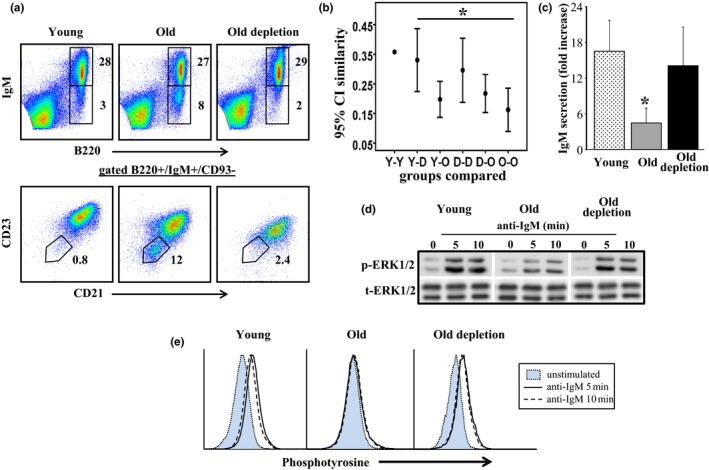
Rejuvenated B cells in old mice mount young‐like population dynamic, repertoire, and responses. Splenic B cells from young, old, and old hCD20Tg treated for B‐cell depletion and reconstituted the peripheral B‐cell compartment (old depletion) were purified. (a) Cells were stained for the indicated surface markers and analyzed by flow cytometry using gates as specified. (b) Ig heavy‐chain repertoires were determined by high‐throughput sequencing. The Morisita similarity index between every two repertoires was calculated. Shown are the 95% confidence intervals of these similarity indices within and between groups of mice: Y—young, O—old, D—old, B‐cell‐depleted mice. (c) B cells were cultured in vitro in the presence or absence of LPS and supernatants were analyzed for secretion of IgM by quantitative ELISA. Results shown are expressed as fold increase (relative to unstimulated cells) and are mean ± *SE* of six mice. (d) B cells were stimulated with anti‐IgM for the indicated time intervals, lysed and analyzed for pERK by Western blotting. The results shown are representative of four mice from each group. (e) B cells were stimulated with anti‐IgM for the indicated time intervals and analyzed for phosphotyrosine by flow cytometry. Shown are representative of four mice from each group

We have next studied the reconstituted B‐cell population. Old hCD20Tg mice were treated for B‐cell depletion and analyzed 50–60 days later, when B‐cell numbers in blood reached ≥90% of the numbers in untreated mice (this group is termed "old‐depleted"). We have first analyzed changes in splenic population dynamic imposed by B‐cell depletion. As shown earlier, with aging there is an accumulation of antigen experienced memory (expressing non‐IgM BCR) B cells (Guerrettaz, Johnson, & Cambier, 2008), as well as pro‐inflammatory "age‐associated B cells" (ABCs) (Ratliff, Alter, Frasca, Blomberg, & Riley, 2013; Rubtsov et al., 2015). The results in Figure 2a show that both populations (antigen experienced B220+/IgM−, and ABCs B220+/IgM+/CD93−/ CD23−/CD21−) were eliminated in mice treated for B‐cell depletion and replaced with newly generated naïve IgM + population. To assess the recovery of repertoire diversity after B‐cell depletion, we performed IgH amplification and deep sequencing from splenic DNA of individual mice that were either young (Y), old (O), or old‐depleted (D). For each pair of repertoires, either within or between groups, we calculated the Morisita similarity index. Figure 2b shows the mean and 95% confidence intervals of the Morisita similarity index for each type of pairing (Y‐Y, Y‐O, etc.). We found that the depleted repertoires are more similar to those of the young than to those of old mice, though this did not reach significance. Additionally, the within‐group similarity was higher in the depleted than in the old group and closer to that of the young. The lowest similarities were within the old group or between old and other repertoires (Figure 2b). We interpret this as showing that the reduced repertoire diversity in old mice, which probably reflects unique clonal expansions, makes each old repertoire very different from the others, while the young repertoires, and to a somewhat lesser extent the depleted group repertoires, are all very diverse and hence all have a similar structure.

We have next determined the cellular responsiveness of purified splenic B cells from each group to immunogenic stimuli in vitro (Figure 2c‐e). In responses to LPS, we found that IgM secretion in cultures of purified B cell from old mice was significantly (3–4 fold) lower than that found in cultures of B cells from young mice. In contrast, the amount of IgM secreted in cultures of B cells of old‐depleted mice was significantly higher and reached the level found in B‐cell cultures from young mice (Figure 2c, *p* < 0.05).

Biochemical analysis upon BCR ligation revealed that ERK phosphorylation (Figure 2d) and total tyrosine phosphorylation (Figure 2e) were significantly reduced in B cells from old mice relative to those of B cells from young mice. In contrast, ERK phosphorylation and total tyrosine phosphorylation in B cells from old‐depleted mice were profoundly increased relative to that of B cells from old mice and were not different from those of B cells from young mice (Figure 2d,e). We conclude that the reconstituted B cells in old mice mount young‐like cellular responses to immune stimuli in vitro.

### 3.3 Reconstituted B cells in old B‐cell lymphoma patients treated with rituximab display young‐like B‐cell population dynamics, antibody repertoires, and responsiveness

**Figure 3:**
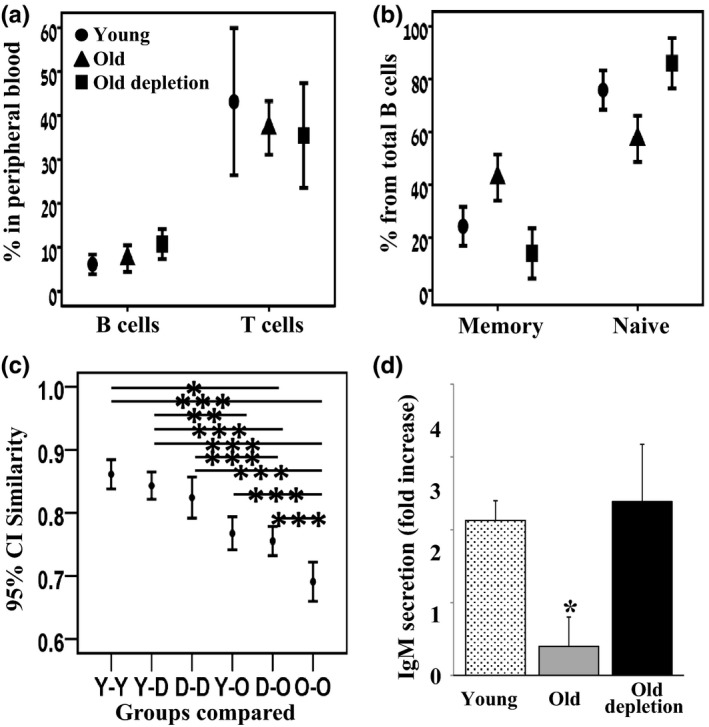
Rejuvenated B cells in old lymphoma patients mount a young‐like population dynamic and function. Peripheral blood samples from young, old, and old‐depleted (lymphoma patients) were collected. (a + b) Cells were analyzed by flow cytometry for frequencies of B (CD19+) and T (CD19−/CD5+) lymphocytes (a) and for memory (CD19+/CD27+) or naïve (CD19+/CD27−/IgD+) B cells (b). Results from young (*n* = 14), old (*n* = 34), and old‐depleted (*n* = 18) patients are expressed. (c) IgH repertoire in peripheral blood was determined by CDR3 spectratyping using DNA extracted from peripheral blood mononuclear cells. The Morisita similarity index between every two spectratypes was measured. Shown are the 95% confidence intervals of these similarity indices within and between groups of patients: Y—young, O—old, D—old, B‐cell‐depleted patients. Statistical significance of differences between groups was calculated by ANOVA. (d) B cells were purified and cultured in vitro in the presence or absence of LPS. Supernatants were analyzed for secretion of IgM by quantitative ELISA. Results shown are expressed as fold increase (relative to unstimulated cells) and are mean ± *SE* of 5–6 patients

To explore whether B‐cell depletion rejuvenates the peripheral B‐cell compartment in humans, we analyzed B‐cell populations in peripheral blood collected from elderly B‐cell lymphoma patients, previously treated with rituximab (old depletion group). As shown in Figure 3a, frequencies of B (CD19+) and T (CD5+/CD19−) cells were not different between young (*n* = 14), old (*n* = 34), and old‐depleted (*n* = 18) patients. However, the frequency of memory (CD19+/CD27+) B cells in old subjects was significantly high, reaching 40%–45% of total B cells, whereas the frequency of naïve (CD19+/CD27−/IgD+) B cells in these subjects was only 55%–60%. In contrast, in the old‐depleted group, memory B cells represented <20% of the entire B‐cell population, whereas the remaining (>80%) were naïve B cells, a similar distribution to that observed in young, healthy, subjects (Figure 3b).

We have next examined the B‐cell repertoire in individuals from each group (young *n* = 9, old *n* = 18, old‐depleted *n* = 13) using spectratypes (IgH CDR3 length distributions). As in the mouse repertoire analysis (Figure 2), we determined the Morisita similarity index within each group and between groups using SPADE (Figure 3c). The mean similarity between young individual spectratypes was the highest, probably because their spectratypes are all relatively close to a normal distribution. The old‐depleted patient spectratypes were most similar to those of the young and to each other, whereas the spectratypes of old healthy subjects were least similar to those of the young and old‐depleted patients and have the lowest similarity to each other. These results are in agreement with those found in mice and suggest that the restricted repertoire that develops with aging is replaced after B‐cell depletion by a more diverse young‐like repertoire.

Lastly, we determined B‐cell responsiveness to immunogenic stimuli. We purified B cells from peripheral blood of young (*n* = 6), elderly (*n* = 5), and elderly depleted (*n* = 5) patients and cultured the cells in vitro with LPS. Quantification of IgM secretion revealed that B cells from old subjects poorly respond to LPS stimulation, whereas B cells from old‐depleted patients responded as vigorously as B cells from young subjects (Figure 3d, *p* < 0.05), suggesting that in rituximab‐treated patients, the reconstituted B cells mount young‐like immune functions.

### 3.4 Responsiveness of old‐depleted mice to immunological challenges

**Figure 4:**
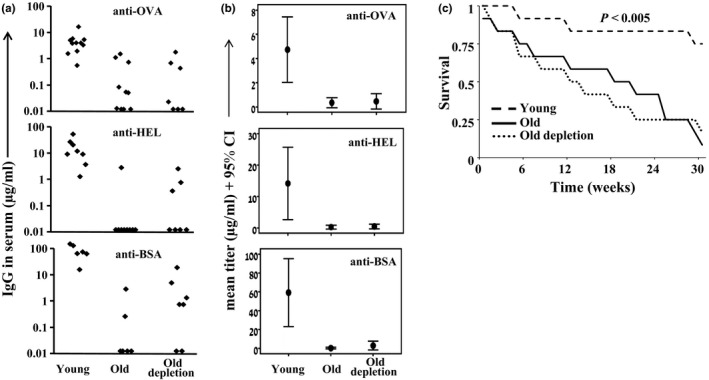
B‐cell rejuvenation does not confer enhanced immunity in old mice. (a, b) young, old, and old depletion (hCD20Tg) (65 days after depletion) mice were immunized intraperitoneally with OVA, HEL, and BSA emulsified in aluminum hydroxide. Mice were bled 14 days later, and anti‐OVA, anti‐HEL, and anti‐BSA IgG antibody titers were determined by ELISA. Shown are titers for individual mice (*n* = 6–12 in each group) (a) and group mean with 95% CI (b). (c) Young (*n* = 12), old (*n* = 12), and old depletion (65 days after depletion) (*n* = 12) mice were transferred from the SPF facility into a dirty facility and were followed for 30 weeks for survival. Shown is the Kaplan–Meier survival plot for each group. Significance was determined using the log‐rank test

The young‐like cellular functions mounted by reconstituted B cells in old humans and mice prompted us to test whether this can be used to enhance immune responsiveness in aging. In a previous study (Keren et al., 2011), we reported that old‐depleted mice produce more anti‐NP IgG1 in response to NP‐CGG immunization relative to old mice. However, this response was still significantly lower than in young mice and varied profoundly between individual mice (by up to 16 folds). This observation led us to carefully re‐evaluate this paradigm. First, we immunized young, old, and old‐depleted (hCD20Tg) mice with three different protein antigens: ovalbumin (OVA), hen egg white lysozyme (HEL), and bovine serum albumin (BSA). Mice were bled 14 days later, and antigen‐specific IgG antibodies were quantified by ELISA. Figure 4a shows an efficient antibody response to all antigens in young mice, whereas old mice were essentially unresponsive in terms of both IgG titers and number of responding mice. The analysis of antibody production in the old‐depleted group revealed detectable titers in 30%–50% of the mice, but these titers were very poor relative to those of the young mice. Statistical analysis indicated that antibody responses in the old‐depleted group were not different from those observed in the elderly control group, and both were statistically inferior relative to the young group (95% CI, Figure 4b). Next, to test this paradigm in a more clinically relevant setup, we transferred young, old, and old‐depleted mice from our specific pathogen‐free facility to a "dirty" facility and followed their survival for up to 30 weeks. The survival rate of old and old‐depleted mice was not different (*p* = 0.99) and approached 90% by week 30 (Figure 4c), and both were significantly higher (*p* < 0.005) relative to 20% in young mice. In aged‐matched old and old‐depleted mice that were not transferred to the "dirty" facility, the extent of death over this time interval was only 20%–30% (3 out of 11 old‐depleted mice, 2 out of 9 old mice). Based on these findings, we concluded that B‐cell depletion does not increase overall immune responsiveness in old mice, as reflected by response to immunization and survival in a "dirty" environment. While this conclusion differs from our previous one (Keren et al., 2011), we think that the present data, which relies on a large number of mice, several antigens and different immunological challenges, better addresses, and quantifies immune responses of old versus old‐depleted mice.

### 3.5 Elderly B‐NHL patients that were treated with rituximab respond to hepatitis B vaccine (HBV) similarly to healthy age‐matched controls

To test whether B‐cell rejuvenation increases immune competence in elderly people, we performed a clinical study where we evaluated antibody responses to HBV vaccine among elderly B‐NHL patients that were treated for B‐cell depletion by rituximab, relative to healthy age‐matched and young control groups.

#### 3.5.1 Participant characteristics and vaccine administration

Seventy‐seven participants were recruited into the study; 42 elderly B‐cell‐depleted NHL patients, 26 elderly, and 9 young healthy volunteers. Twenty‐nine individuals, including 19 elderly B‐cell‐depleted patients and 10 healthy volunteers (9 elderly and one young), were not vaccinated with HBV. Reasons for nonvaccination in the B‐cell‐depleted group were insufficient B‐cell recovery according the study's criteria at the time of recruitment (*n* = 10) or patient refusal (*n* = 9). The main cause for nonvaccinating in the healthy control groups was refusal (Table S2, Appendix S1, presents participants flowsheet).

Twenty‐three elderly B‐cell‐depleted patients were vaccinated; 22 completed the 3 vaccine doses and were therefore evaluable for response, one patient received only two vaccines and was omitted from the response analysis. Seventeen healthy elderly individuals and eight young individuals completed all three doses and served as the control groups.

The median age at vaccination for the B‐cell‐depleted NHL group approached 65 years (range 55–79 years), versus 63 years for the elderly healthy group (range 57–65) (*p* = ns) and 32 years (range 28–35) for young healthy cohort. Median time from last rituximab treatment (for rituximab exposed, NHL patients only) was 38 months (ranging from 14–56 months). All 141 vaccine administrations went uneventfully and were not accompanied by any grade 2–4 toxicities.

#### 3.5.2 Vaccine efficacy

**Figure 5:**
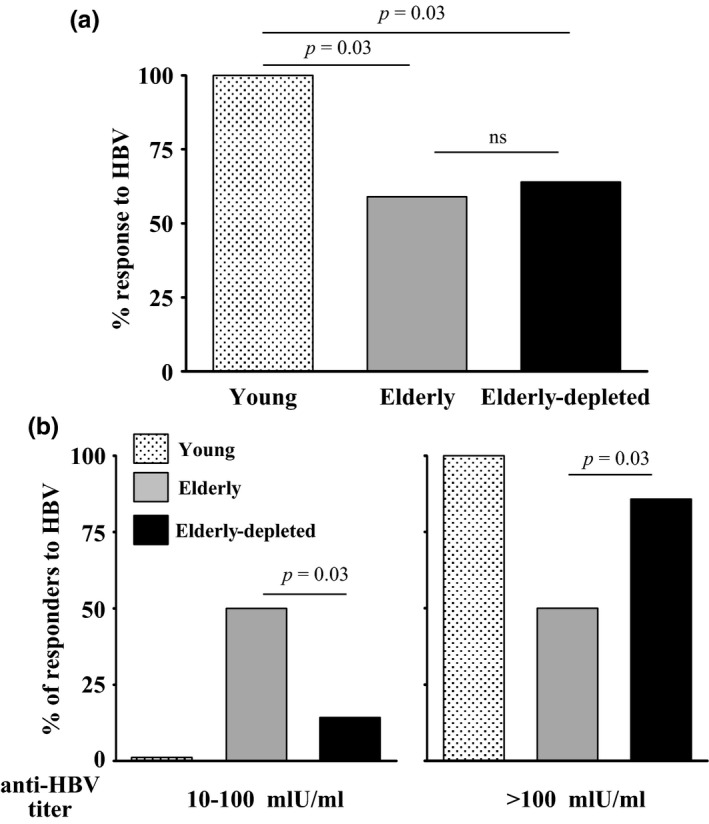
Humoral anti‐HBV response obtained in elderly B‐cell‐depleted patients relative to elderly and young healthy volunteers. Individuals from the indicated groups were vaccinated with HBV, and anti‐HBV antibody responses were determined as detailed in the methodology section. (a) Graph depicts percent of responders to HBV (antibody titers of ≥10 mIU/ml) for each group. (b) Graph depicts distribution of HBV responders developing titers of 10–100 mlU/m and those developing titers of >100 mlU/ml

The overall anti‐HBV response in the elderly depleted cohort was not superior to that developed in the old control group and both were equally inferior relative to the young control cohort (*p* = 0.03) (Figure 5a). Thus, only 14 out of 22 (64%) elderly depleted patients and 10 out of 17 (59%) old control individuals responded to HBV vaccination (*p* = ns), whereas among the young healthy subjects, the response rate approached 100% (Figure 5a). Yet, analysis of anti‐HBV antibody titers revealed that >85% of the elderly depleted patients who did respond to the HBV vaccine developed antibody titers higher than 100 mIU/ml, in contrast to only 50% of the responders among the old healthy subjects and 100% of the young vaccinated subjects (Figure 5b). No statistically significant differences in age, sex, or time from last rituximab dose, between responders and nonresponders, were found. Overall, these results suggest that, while B‐cell depletion does not improve the response rate to HBV vaccine in aged individuals, it may result in enhanced antibody titers among responders, which are associated with long‐term immunity (Hattum, 1995).

## 4 DISCUSSION

An irreversible, age‐related decrease in production of naïve B cells in the BM was suggested to account for the increased risk of infections observed in elderly subjects (Gibson et al., 2009; Guerrettaz et al., 2008; Lescale et al., 2010; Linton & Dorshkind, 2004; Riley, 2013; Rossi et al., 2005). In recent years, intervention approaches relying on removal of "old" tissues or cells demonstrated rejuvenation capacity in mouse models of aging tissues (Baker et al., 2011; Chang et al., 2015; Jeon et al., 2017). In accordance with this, we have shown that B‐cell depletion reactivates B lymphopoiesis in old mice (Keren et al., 2011), thereby proposing a mechanism to restore B‐cell responses in aging. Indeed, our current findings suggest that B‐cell recovery following depletion is not just a "recapturing" process, which returns B cells to the same stage they have been in before being exposed to depletion, but a rejuvenation process, in which the B‐cell repertoire becomes younger both phenotypically and functionally, resulting from de novo B lymphopoiesis. This rejuvenation is observed in both aged experimental mice and in elderly humans. We proposed that B lymphopoiesis in aging is suppressed by the accumulated antigen‐ experienced B cells in the periphery (Keren et al., 2011). Recent studies proposed that this cross‐talk is mediated by TNFα, being produced by "age‐associated B cells" (ABCs) which accumulate in aged mice and humans (Ratliff et al., 2013; Rubtsov et al., 2015). We show here that this population is removed by B‐cell depletion and replaced by naïve newly generated B cells, thereby suggesting that removal of suppressive effects mediated by ABCs supports the reactivation of B lymphopoiesis in the BM, as we have shown previously (Keren et al., 2011).

The B‐cell rejuvenation strategy that we show here confers a proof of concept, that such an approach is physiologically applicable. A rejuvenated immune system would potentially respond to new and/or evolving pathogens, thereby may decrease the risk of infections, an objective that is often unachieved in elderly subjects. Such enhanced immunity may also benefit from a rejuvenated B‐cell population dynamics and increased numbers of naïve newly generated B cells that are more responsive to immunogenic stimuli due to expression of competent TLR and/or BCR molecules and more efficient signaling pathways. While we have shown that differences in population dynamic and BCR signaling do occur after depletion, it is yet to determine whether changes in expression of BCR and TLR are also accompanying B‐cell rejuvenation. Interestingly, plasma cells are not targeted by anti‐CD20 antibodies and are not depleted (Taylor & Lindorfer, 2008). These cells may account to the low level of antibody titers in the old immunized mice. In humans, long‐lived plasma cells are thought to maintain high level of specific antibodies in rituximab‐treated patients for several months after treatment (Cambridge et al., 2006; Ferraro, Drayson, Savage, & MacLennan, 2008).

The B‐cell depletion approach is an approved therapy for lymphoma patients and for some autoimmune diseases (Salles et al., 2017; Schioppo & Ingegnoli, 2017). A recent article has shown that B‐cell depletion in systemic sclerosis patients not only promotes remission but also temporarily reverses the disease‐restricted B‐cell repertoire due to new B‐cell generation (Bourcy, Dekker, Davis, & Nicolls, 2017). The present study proposes that this rejuvenation may be also induced in elderly population, where B lymphopoiesis is suppressed.

However, rejuvenation of the B‐cell compartment in itself is still insufficient to restore full immune responsiveness in aging. Thus, B‐cell depletion that is followed by rejuvenation failed to increase the response rate to hepatitis B vaccine in aged humans, or to prolong the survival of old mice exposed to dirty environments. These findings suggest that the in vivo immune response evoked post‐B‐cell depletion, at least to these stimuli, may still be suboptimal, due to concurrent, age‐related impairments in other essential components of immunity (Linton & Dorshkind, 2004; Nikolich‐Žugich, 2008). Indeed, age‐related defects have been reported in T lymphocytes (Goronzy, Fang, Cavanagh, Qi, & Weyand, 2015; Nikolich‐Žugich, 2008), monocytes dendritic cells (DCs) (Agrawal & Gupta, 2011), monocytes (Metcalf et al., 2017), and NK cells (Gounder et al., 2018). Thus, although B‐cell depletion provides a proof of principle for a rejuvenation approach in the immune system, it is insufficient to completely restore immune competence, since all other essential counterparts of cellular immunity are still “old". The fact that all B‐cell‐depleted elderly patients had also received chemotherapeutic agents that affect T‐cell function may also contribute to this inadequate responsiveness to HBV vaccine. Yet, the proportion of elderly individuals that attain anti‐HBV titers that are >100 mIU/ml and are associated with a high likelihood for a long‐term immunity (Hattum, 1995) was higher among old‐depleted patients. A similar phenomenon was also observed in old‐depleted mice that were immunized with BSA, OVA, and HEL, although the difference was not statistically significant (Figure 4) (Keren et al., 2011). It is therefore possible that these enhanced antibody titers reflect, to some extent, the improved functioning of the rejuvenated B cells, as revealed by the restored activation of signaling cascades. Lastly, in our human vaccination study the number of participating patients was relatively low resulting from the inclusion criteria. Although our conclusions are well supported by the statistical analysis, it is possible that the relative small size of vaccinated patient groups may pause a study limitation.

In summary, the current study proposes a new paradigm to restore immunity in aging throughout a physiologically relevant mechanism. The fact that similar observations were found in elderly humans and old mice suggest that this paradigm should be explored for anti‐aging interventions. Further studies, looking at B‐cell rejuvenation approaches, but also at strategies to rejuvenate other cellular immunity components, are warranted.

## CONFLICT OF INTEREST

None declared.
