# Supplementary material for: Depletion of B cells rejuvenates the peripheral B‐cell compartment but is insufficient to restore immune competence in aging
Source: Aging Cell. 2019 May 6;18(4):e12959. doi: 10.1111/acel.12959 (PMC6612643; doi:10.1111/acel.12959)
Supplement: Supplementary file 1 [file ACEL-18-e12959-s001.pdf]

Supplementary methods

Mice, B cell depletion and immunizations

In some experiments, mice were immunized intra-peritoneally with a mixture of ovalbumin (OVA, 10μg/mouse), hen egg-white lysozyme (HEL, 10μg/mouse) and bovine serum albumin (BSA, 10μg/mouse) precipitated in aluminum hydroxide. Mice were bled 14 days later, and sera were analyzed for specific antibodies by ELISA. In other experiments, mice were transferred from the SPF room to a conventional "dirty" room and were followed for survival. The mice were housed at the animal facility of the Faculty of Medicine, Technion, and all studies were approved by the institute's committee for the supervision of animal experiments.

B-cell purification, stimulations and flow cytometry

B cells from mouse spleens or from human peripheral blood were isolated using EasySep magnetic beads (STEMCELL Technologies). Purified B cells (2x106 in 500μl medium) were stimulated with LPS (25μg/ml), and supernatants were collected after 96 hr and analyzed for IgM secretion by ELISA. In some experiments, mouse B cells were stimulated with F(ab')2 of goat anti-mouse IgM (Jackson ImmunoResearch) 10 μg/ml and analyzed for tyrosine phosphorylation by flow cytometry (using anti-phosphotyrosine-Alexa Flour 647, clone PY20, Biolegend), or lysed and analyzed for ERK phosphorylation by western blotting (using rabbit anti-phospho-ERK1/2 and rabbit anti-ERK1/2 from Cell Signaling) as described[1]. B cell populations in mouse bone marrow or in human peripheral blood were analyzed by flow cytometry as described[2]. Antibodies used for mouse cells were anti-B220-PB (RA3- 6B2), anti-CD19-BV605 (6D5), anti-CD93-APC (AA4.1), from BioLegend, anti-CD43 (S7) from BD Bioscience and goat α-mouse IgM-FITC, μ chain specific, from Jackson ImmunoResearch. Antibodies used for human cells were anti-CD19-APC

(HIB19), anti-CD27-PB (O323), anti-CD10- PerCP/Cy5.5 (HI10a) from Biolegend, anti-IgD-Bio (Cat 555777) anti-CD5-PE (L17F12) from BD bioscience. Data for four- and- five-color analysis were collected on a FACSCyAn ADP (Beckman Coulter Inc.) and analyzed using the FlowJo software (Tree Star).

Antibody quantification

Quantification of mouse or human IgM in supernatants and quantification of IgG specific for OVA, HSA or BSA in mouse sera were performed by ELISA[3].

Immunoglobulin heavy chain repertoire analysis

Mouse splenic B cell IgH repertoires were investigated by high throughput sequencing. DNA was extracted from splenic B cells of 3 old (>20 month), 2 young and 2 B cell-depleted Balb/C mice using DNA purification kit (QIAGEN). Mouse Ig heavy and light chains were amplified by semi-nested PCR and sequencing as detailed below.

Human peripheral blood repertoires were investigated by spectratyping using DNA extracted from peripheral blood mononuclear cells (Qiagen, Crawley) as described[4]. The CDR3 region of the rearranged IgH was amplified using the IgH Gene Clonality Assay (Invivoscribe) and products were run by capillary electrophoresis (ABI PRISM Genetic Analyzer) according to manufacturer instructions (Applied Biosystems). The results were analyzed using GeneScan (Applied Biosystems) to determine the peak sizes for each main peak in the spectratype. The peaks at 3-bp intervals were further analyzed as described below.

PCR, sequencing and mouse Ig repertoire determination

IgH and IgL repertoires were determined by PCR and sequencing. For the mouse Ig heavy, kappa and lambda chains we used three separate primer sets of 8, 8, and 3 forward

primers respectively within the FR1 region, and 2 reverse primers for each chain type [5]. The 5′ sequence of the primer (IDT, HPLC grade) contained an adaptor that is specific for the 454-FLX emulsion PCR, followed by a 10bp multiplex identifier (MID) tag (supplementary Figure 2).

PCR was performed with the XP Thermal Cycler (BioER). Each reaction contained 10-100ng of genomic DNA as a template, 0.6-1.2mM dNTP (Fermentas), 0.4uM primers, 1mM MgCl2 and 1-2U of proofreader Taq polymerase (AB gene or Finnzyme). According to the manufacturer, the polymerase error rate is of an order of magnitude of 10-7bp. Each round of PCR contained an initial denaturation step at 98°C for 3 minutes. Denaturation and extension were carried out at 94°C for 60s and 72°C for 90s throughout, respectively. The annealing temperature in the first 5 cycles was 52.5°C for 60s, followed by 5 cycles at 52°C and 25 additional cycles at 51.5°C. The second PCR round was performed by the same cycling program as in the first round. PCR products were separated by electrophoresis on 2% agarose gel containing ethidium bromide. Sharp bands of around 400bp were cut out of the gel, and DNA was cleaned by QIAquick gel extraction columns (Qiagen) according to the manufacturer's protocol. Two distinct rounds of high-throughput sequencing were performed the 454 flex titanium instrument by Dyn Diagnostics, Israel. For each round, DNA concentration and the quality of cleaned PCR products were measured by a NanoDrop 3300 Fluorospectrometer (Thermo Scientific) using Quant-iT™ PicoGreen® dsDNA Reagent (Invitrogen) according to the manufacturer's protocol. Sequencing data were processed, annotated, assigned to clonally-related groups, and analyzed as described [6].

<u>Analysis of BrdU Incorporation</u>

Mice were analyzed for BrdU incorporation 65 days after depletion, when reconstitution of the peripheral compartment was completed [7]. BrdU labeling in vivo, staining and analysis

was as previously described [7]. The data on total B cell numbers, the fraction of cells in each B cell subset, and the fraction of BrdU-positive cells within each subset were recorded and analyzed, employing a unique mathematical model, as described below.

Mathematical modeling of B cell development population dynamics and B cell depletion

The model (Supplementary Figure 3A) is fully described by equations 1-5 below. The data did not include pro- and pre-B subpopulations; hence we included in the model a single population that combined both subsets, B_{oe}, to represent the cells in the pro- and pre-B cell subsets that eventually differentiate to the immature subset. B_{oe} cells differentiate into the immature subset with a constant rate denoted by δ_{oe} (per six hours, which is our simulation time unit). The B_{oe} subpopulation is renewed from previous subsets at the constant rate s. This subpopulation proliferates after passing heavy or light chain rearrangements with rate γ (Supplementary Figure 3A and equation 1). On the other hand, the data included the mature recirculating B cells subset in the BM, so we added this subset to our mathematical model (Supplementary Figure 3A and equation 3). The numbers of cells in the immature, mature recirculating, transitional and splenic mature subsets are represented by the variables B_i, B_{Mrec}, B_t and B_{Mspl} respectively (Supplementary Figure 3 and equations 2-5). The mature recirculating subpopulation includes plasma cells that competes with pre-pro-B cells for survival niches [8], therefore the carrying capacity parameter, *k*, that limits the proliferation rate of the pro-pre-B cell subpopulation, includes the total number of cells in the pro-pre-B and the mature recirculating compartments (equation 1).

Immature B cells either differentiate to BM mature cells at rate δ_{i_re} or immigrate from the BM to the spleen and differentiate to transitional B cells at rate δ_{i_t} (equations 2-4). Transitional B cells differentiate to splenic mature B cells at rate δ_t (equations 4-5). After

their maturation splenic mature B cells can go back to the PB and then to the mature recirculating population in the BM. The inflow of mature B cells from the spleen to the mature (recirculating) population in the BM is represented by the parameter ϕ_s. The opposite direction is represented by the parameter ϕ_{BM} (equations 3, 5). The death rates are denoted by μ_i, μ_t ,μ_{rec} and μ_m for B_i, B_t and B_{Mrec},, respectively. The exit from the splenic mature B cell, including death or transition to other organs, is denoted by $\varepsilon_{spl.}$ Based on previous studies, it is assumed that the transitional subpopulation is not cycling [9,10]. Thus, the basic model is described by the following equations.

$$1.\ \frac{dB_{oe}}{dt} = s + \{\gamma[1 - \frac{(B_o + B_{Mre})}{K_o}] + \delta_{oe}\}B_{oe} + \delta_r B_i$$

$$2.\ \frac{dB_i}{dt} = \delta_{oe}B_{oe} - B_i(\mu_i + \delta_{i\_t} + \delta_r + \delta_{i\_re})$$

$$3.\ \frac{dB_{Mre}}{dt} = \delta_{i\_re}B_i + \phi_s B_{Mspl} - (\mu_{re} + \phi_{BM})B_{Mre}$$

$$4.\ \frac{dB_t}{dt} = \delta_{i\_t}B_i - B_t(\mu_t + \delta_t)$$

$$5.\ \frac{dB_{Mspl}}{dt} = \delta_t B_t + \phi_{BM} B_{Mre} - (\phi_s + \varepsilon_{spl})B_{Mspl}$$

To model BrdU labelling, the modeled subsets were further divided into labeled and unlabeled B cell populations. Cells in each unlabeled compartment move to the corresponding labeled subset upon dividing (Supplementary Figure 3B). When an unlabeled cell divides during the labeling period, it leaves the unlabeled subpopulation. We neglected BrdU toxicity (and thus assigned the same death rates to labeled and unlabeled cells), as in our previous work; we found that when the BrdU experiment is very short – as in this case, only 7 days – the latter assumption holds.

Supplementary Figures

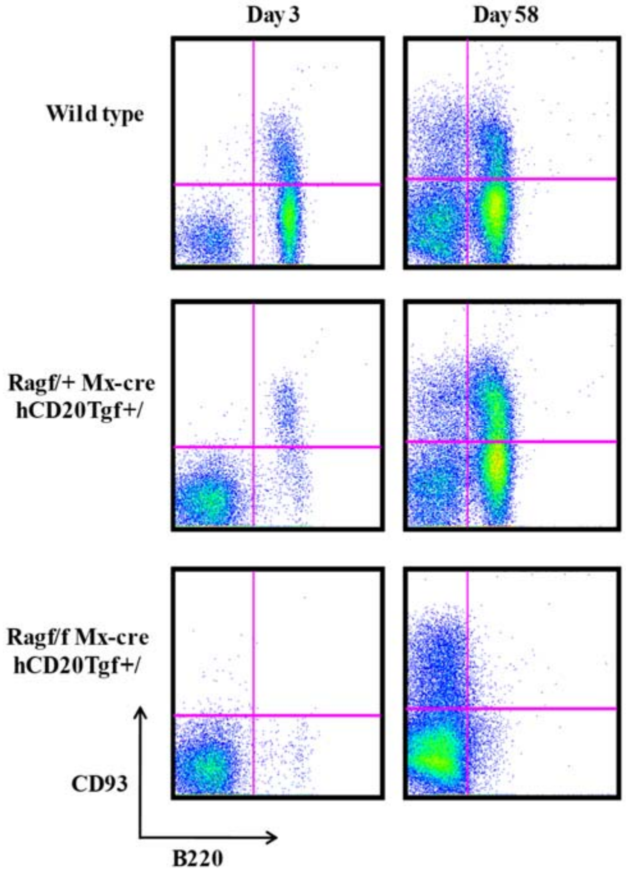

Supplementary figure 1: Following B cell depletion in old mice, reconstituted B cells are derived from de-novo B lymphopoiesis. Mice with the indicated genetic background were injected with poly(I)(C) to ablate RAG-2-floxed alleles, followed by injection of anti-hCD20 antibodies to deplete peripheral B cells. At the indicated time intervals after B cell depletion, peripheral blood samples were collected, and white blood cells were stained for the indicated surface markers. As shown, in RAG-$2^{fl/fl}$/Mx-cre/hCD20Tg (bottom panels) there was no recovery of the B lineage (revealed by the nearly complete absence of B220+ cells in blood on day 58). In contrast, complete reconstitution was observed in RAG-$2^{fl/+}$/Mx-cre/hCD20Tg (middle panels) with about 50% of the cells expressing the CD93 marker indicating the cells were newly generated in the BM. Shown are representative results from 4 mice in each group.

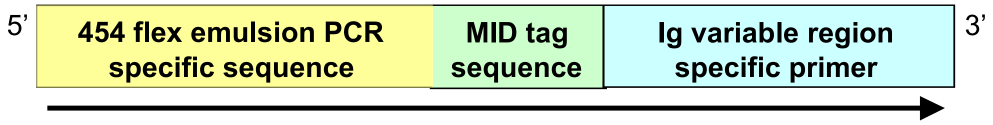

Supplementary figure 2: PCR primer construction.

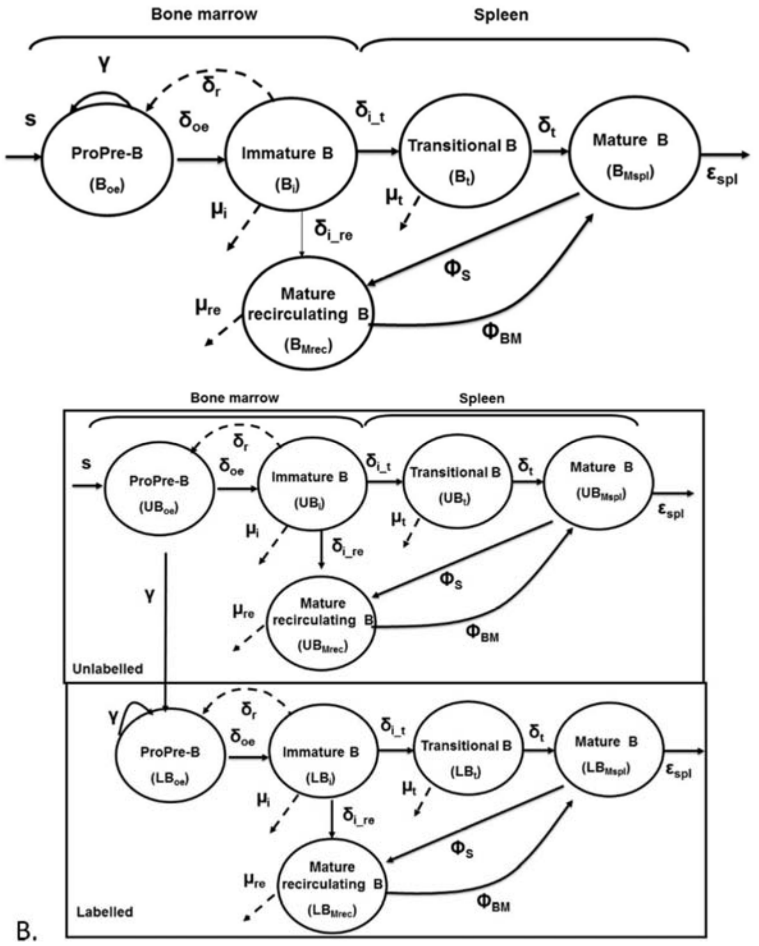

Supplementary Figure 3. Model of B cell populations in the bone marrow and spleen. (A) The model of maturing B-cell populations in the bone marrow and spleen is shown. All population processes – differentiation, proliferation and death – are described by arrows. The rate of each process is given near the corresponding arrow. S – Source of B lineage precursors, δ – differentiation rate, μ – mortality rate, γ – proliferation rate, Φ – flow rate. (B) The model for the labeling dynamics of developing B-cells in the bone marrow assumes that cells in the unlabeled pro and pre-B cell compartment move to the corresponding labeled compartment upon dividing. The model also assumes that each labeled cell remains labeled for the duration of the experiment.

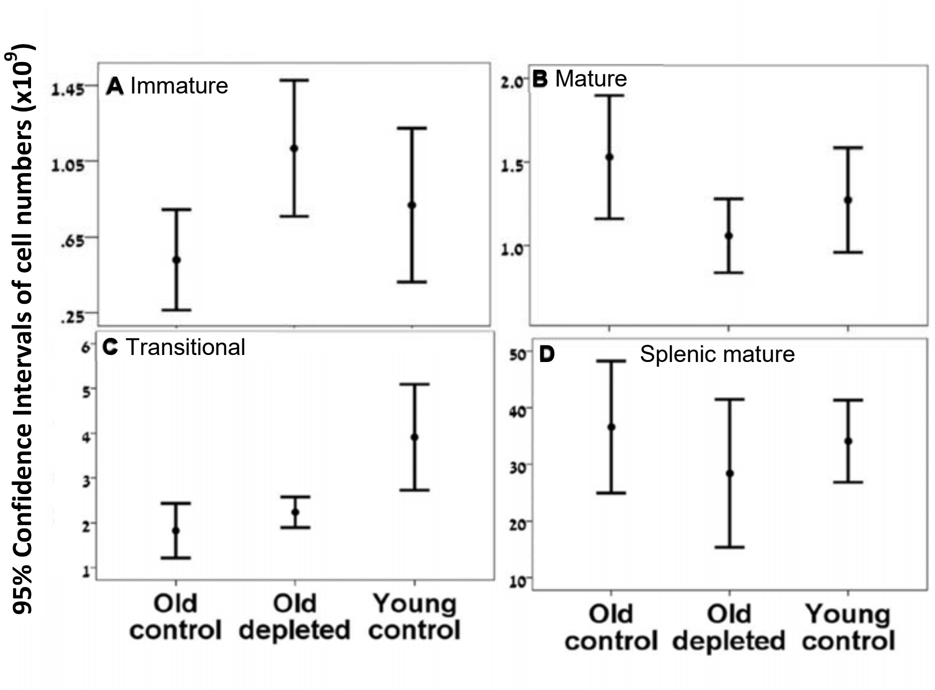

Supplementary Figure 4: Comparing the mean total cell numbers in each B cell subset between old and young control and old B cell depleted mice. Mice were analyzed for BrdU incorporation 65 days after depletion. The mean total cell number in the **immature** B cell subpopulation (A) was significantly lower in old mice than in old depleted mice (P value<0.05). The mean total cell number in the **mature recirculating** B cell subpopulation in the BM (B) was higher in old mice than in young and old depleted mice (P value=0.001 and 0.098, respectively). B cell depletion had no effect on the total number of **transitional B cells** (C); however, transitional B cell numbers seem to be affected by age. Depletion had no effect on the total number of **splenic mature B cells** (D). Thus, depletion reduces the number of mature recirculating B cells in the BM, freeing more niches for new B cell development.

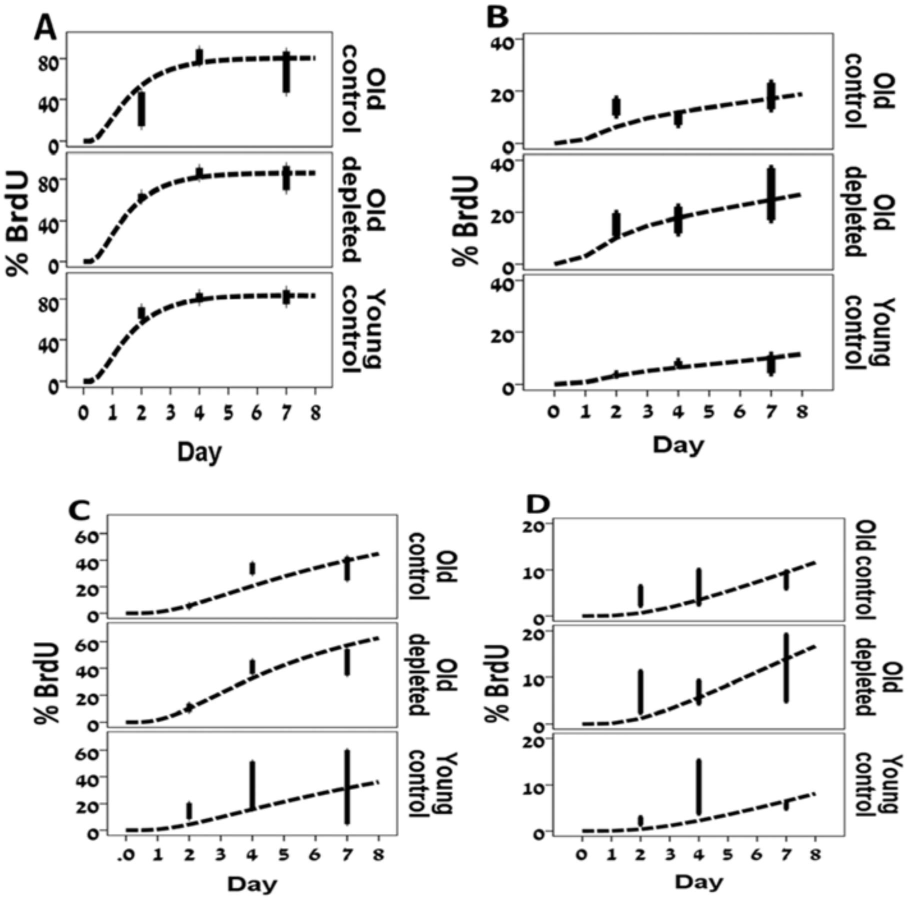

Supplementary Figure 5: BrdU labeling kinetics obtained by a simulation of the B cell development model using the parameter value set that gave the best fit to the data. Simulations results are shown as lines, along with the data (symbols); at least five mice were sacrificed per time point. (A) **Immature B cells**: On day 2, the percentages of BrdU+ cells in the immature B cell subset in old mice was significantly lower than the same percentage in the other mouse types (P value<0.01), although later the percentages became similar. (B) **Mature recirculating B cells**: on day 2, the percentages of BrdU+ mature recirculating B cells in old mice were significantly higher than the same percentage in the young mice (P value<0.01), regardless of depletion. In old B cell depleted mice, BrdU+ mature recirculating B cells and increased on day 7, at which point this fraction was higher in old depleted mice than in young control mice (P value<0.01). (C) **Transitional B cells**. (D) **Splenic mature B cells**.

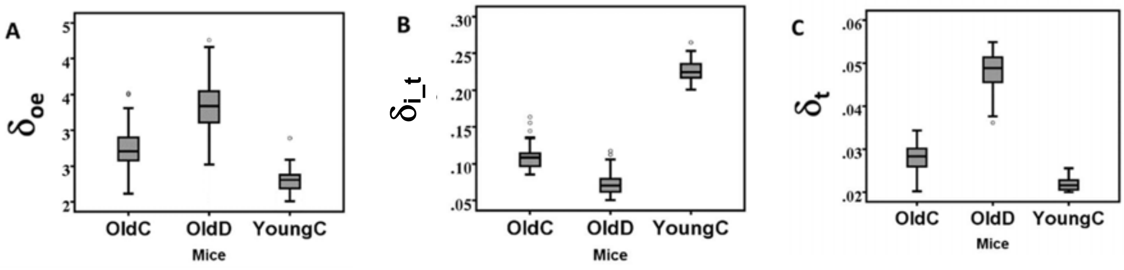

Supplementary Figure 6: Box Plots of parameter value distributions for each rate parameter that was found to significantly (or largely) differ between the simulations of young, old control and depleted mice. δ_{oe} is the differentiation rate from the proliferating subpopulation into the immature subset; δ_{i_t} is the rate of immature cell differentiation into transitional B cells, and δ_t is the rate of transitional B cell differentiation into the splenic mature B cell subset.

Supplementary Table 1: The 95% confidence intervals for parameter values obtained in simulations of labeling kinetics of control and depleted B cells.

Rate (1/6h) [a]	Old control		Old depleted		Young control	
	CI 95% [b]		CI 95%		CI 95%	
	Lower Bound	Lower Bound	Lower Bound	Lower Bound	Lower Bound	Lower Bound
**S (x 10^5 cells)**	1.05	2.02	1.01	1.80	0.81	1.36
γ	.60	.99	.55	1.00	.71	.98
δ_{oe}	.21	.35	**.25**	**.43**	.20	.29
μ_i	.11	.28	.15	.22	.10	.29
δ_{i_t}	.09	.16	.05	.12	**.20**	**.26**
δ_{i_re}	.04	.04	.04	.04	.04	.04
μ_t	.001	.014	.001	.010	.001	.003
δ_t	.02	.03	**.04**	**.05**	.02	.03
ϕ_{BM}	.50	.94	.50	1.00	.50	.61
μ_{re}	.03	.10	.002	.10	.02	.07
ϕ_s	.030	.038	.020	.038	.035	.040
ε_{spl}	.001	.007	.004	.014	.001	.003

[a] The time unit of the simulations was 6 hours.

[b] Shown in bold with gray highlighting are the cases in which the value of the indicated parameter (row) differs between the indicated mouse type (column) and the other mouse types.

Supplementary Table 2: Causes for "screen failure" for vaccination.

	Study group Elderly<br><br>NHL pts	Control Elderly<br><br>Healthy subjects	Control young<br><br>Healthy subjects
**Recruited**	42	26	9
**Have not been vaccinated**	19	9	2
**Failed study criteria**	3		
Low IgG levels	6	NR	
Refused to receive vaccination	10	9	1
**Vaccinated (n)**	23*	17	8

*One patient was not included in the final analysis, having received 2 vaccine doses only

REFERENCES

1. Novak R, Jacob E, Haimovich J, Avni O, Melamed D. The MAPK/ERK and PI(3)K Pathways Additively Coordinate the Transcription of Recombination-Activating Genes in B Lineage Cells. *The Journal of Immunology*. 2010;185(6):3239-3247.
2. Keren Z, Naor S, Nussbaum S, Golan K, Itkin T, Sasaki Y, Schmidt-Supprian M, Lapidot T, Melamed D. B-cell depletion reactivates B lymphopoiesis in the BM and rejuvenates the B lineage in aging. *Blood*. 2011;117(11):3104-3112.
3. Seagal J, Leider N, Wildbaum G, Karin N, Melamed D. Increased plasma cell frequency and accumulation of abnormal syndecan-1plus T-cells in Igmu-deficient/lpr mice. *Int Immunol*. 2003;15(9):1045-1052.
4. Michaeli M, Tabibian-Keissar H, Schiby G, Shahaf G, Pickman Y, Hazanov L, Rosenblatt K, Dunn-Walters DK, Barshack I, Mehr R. Immunoglobulin Gene Repertoire Diversification and Selection in the Stomach – From Gastritis to Gastric Lymphomas. *Frontiers in Immunology*. 2014;5(264).
5. Rohatgi S, Ganju P, Sehgal D. Systematic design and testing of nested (RT-)PCR primers for specific amplification of mouse rearranged/expressed immunoglobulin variable region genes from small number of B cells. *Journal of Immunological Methods*. 2008;339(2):205-219.
6. Tabibian-Keissar H, Hazanov L, Schiby G, Rosenthal N, Rakovsky A, Michaeli M, Shahaf GL, Pickman Y, Rosenblatt K, Melamed D, Dunn-Walters D, Mehr R, Barshack I. Aging affects B-cell antigen receptor repertoire diversity in primary and secondary lymphoid tissues. *European Journal of Immunology*. 2016;46(2):480-492.
7. Shahaf G, Zisman-Rozen S, Benhamou D, Melamed D, Mehr R. B Cell Development in the Bone Marrow Is Regulated by Homeostatic Feedback Exerted by Mature B Cells. *Frontiers in Immunology*. 2016;7(77).
8. Fairfax KA, Kallies A, Nutt SL, Tarlinton DM. Plasma cell development: from B-cell subsets to long-term survival niches. *Semin Immunol*. 2008;20(1):49-58.
9. Allman D, Lindsley RC, DeMuth W, Rudd K, Shinton SA, Hardy RR. Resolution of Three Nonproliferative Immature Splenic B Cell Subsets Reveals Multiple Selection Points During Peripheral B Cell Maturation. *The Journal of Immunology*. 2001;167(12):6834-6840.
10. Shahaf G, Allman D, Cancro MP, Mehr R. Screening of alternative models for transitional B cell maturation. *International Immunology*. 2004;16(8):1081-1090.
